# The health of public transport drivers during COVID-19

**DOI:** 10.47626/1679-4435-2023-992

**Published:** 2023-04-18

**Authors:** Luis David Berrones-Sanz

**Affiliations:** 1 Colegio de Ciencia y Tecnología, Universidad Autónoma de la Ciudad de México, Ciudad de México, Mexico

International literature shows that professional drivers compared with workers in general have a higher prevalence of chemical, physical, mechanical, and biological risks. So, it is not surprising that in Mexico - due to the number of deaths, illnesses, and occupational disabilities^1^ - road transport workers are the second group in terms of risks associated with their profession. Likewise, since their activity makes them come into contact with dozens or hundreds of passengers per day, passenger transport drivers have increased their biological risks concerning COVID-19 because they are prone to the dispersion and transmission of SARS-CoV-2.

Thus, we decided to study the working and health conditions of public transport drivers during the pandemic. To this end, statistical monitoring has been conducted and surveys and interviews have been performed with various groups of transport workers, including drivers of pedicabs, taxis, minibuses, buses, and the Mexico City subway (known as Metro). Previous studies^2^ have reported job insecurity for drivers; however, it is also known that drivers who work for public bodies have the best social benefits and, therefore, the best working conditions.

In addition, the pandemic increased this difference since the influx in the different types of transport fell by 60% to 70%^3^ and, therefore, the income of independent drivers fell proportionally. Although the number of infections varies between the types of transport, the proportion currently reaches up to 40% and the number continues to rise. For example, 24.5% of subway drivers have become ill with COVID-19, even though they are isolated in their driving cabin and their dealings with users is relatively little. In this way, compared with the population of Mexico City, these drivers have a 1.7 (95% CI, 1.53-1.89) times greater probability of infection.

Given the evidence of super-spreading events of diseases through transport systems,^4^ it is necessary to reinforce social distancing measures and, although demand has decreased, governments should subsidize transport systems to maintain or increase service frequency. In this way, the line capacity of the transport systems will be increased, there will be a lower concentration of users and, therefore, the risk of infection will be reduced.

However, to protect both drivers and passengers, prevention and distancing measures must not only be reinforced in public transport by avoiding cash payments and separating drivers with plastic tape barriers; the role of professional drivers, whose activities have a strong impact on public health, must be reconsidered since they are inserted in an alienating work process, exposed to risks and demands and, consequently, to the physiological and psychological manifestations that determine living and working conditions in a precarious situation.

In this respect, it is essential to investigate the professional drivers of public transport and, thus, to ensure that they achieve mastery of their work, get to know and identify the objects, the means, the organization and the activities they carry out and, finally, that they may take action on their health-disease process.

## References

[1] Instituto Mexicano del Seguro Social (2017). Memoria Estadística 2017. Salud en el Trabajo.

[2] Berrones-Sanz L. D, González-Peña E. C (2018). Estado del arte de las condiciones laborales y de salud de los
choferes profesionales [State of the art of the work and health conditions
of the professional drivers]. Red De Investigación En Salud En El Trabajo.

[3] Secretaría de Movilidad (2020). Movilidad durante la emergencia sanitaria COVID-19.

[4] Denphedtnong A, Chinviriyasit S, Chinviriyasit W (2013). On the dynamics of SEIRS epidemic model with transport-related
infection. Math Biosci.

